# In Vitro Analysis of Bonding and Wear Properties of 3D Printed Denture Tooth Materials

**DOI:** 10.7759/cureus.65388

**Published:** 2024-07-25

**Authors:** Sompop Bencharit, Chance A Hunsaker, Christian B Brenes

**Affiliations:** 1 Workman School of Dental Medicine, High Point University, High Point, USA

**Keywords:** wear resistance, bonding strength, denture materials, 3d printing, additive manufacturing

## Abstract

Purpose: While additive manufacturing (3D printing) has recently enhanced removable prosthodontics, the properties of new 3D printed materials are not well understood. This study aims to elucidate the physical properties of these materials, focusing on bonding to a 3D printed denture base material and wear resistance.

Methods: For denture tooth-denture base bonding analyses, the same denture tooth material (Premium Teeth, Formlabs) was used with three denture base-bonding group assignments (n=6 each group) bonded using three protocols: Group A1 was bonded with Lucitone Digital Print-3D Denture Base using the Lucitone Fuse System (Dentsply), Group A2 with Formlabs Denture Base using the Formlabs Denture Base Bonding System, and Group A3 with Formlabs Denture Base using the Ivoclar Ivotion Bonding System (Ivoclar). Specimens were made according to the ISO-TS-19736-2027 standard. A 3D printed tooth mimicking a central incisor was bonded to the denture base and subjected to a palatal load at the incisal region at 90° from the long axis of the tooth until failure. The fracture surface was examined at 10× magnification. ANOVA with α=0.05 was used to determine statistically significant differences. For wear analysis, the same denture base material and bonding system (Lucitone Digital Print-3D Denture Base/Lucitone Fuse System, Dentsply) was used with four denture tooth material group assignments (n=8 each group): Group B1 used Formlabs Premium Teeth, Group B2 used SprintRay High Impact Denture Teeth, Group B3 used Lucitone Digital IPN Premium Tooth, and Group B4 used Ivotion Polymethyl Methacrylate (PMMA) Milled Teeth (Ivoclar). A premolar denture tooth bonded with the denture base was subjected to a chewing simulation cyclic loading of 1,200,000 cycles. Sample failures, vertical wear, and volume loss were documented. ANOVA with α=0.05 was used to determine statistically significant differences.

Results: The fracture load to failure values for A1, A2, and A3 were 175±106 N, 167±46.3 N, and 183±48.9 N, respectively (p=0.95). Most failure characteristics were mixed, except one of A2 was cohesive and half of A3 was cohesive. For cyclic loading, B4 was the only group where all specimens failed within 1,200,000 cycles, while B1, B2, and B3 had four, three, and five sample failures, respectively. Vertical wear was 0.93±0.34 mm, 1.22±0.37 mm, 1.05±0.27 mm, and 0.37±0.02 mm for B1, B2, B3, and B4, respectively (p<0.01). Abrasion volumes were 9.5±3.7 mm³, 12.2±4.7 mm³, 10.6±3.5 mm³, and 2.2±1.3 mm³ for B1, B2, B3, and B4, respectively. Vertical height loss per chewing cycle (μm/cycle) was 0.0022±0.0019, 0.0030±0.0029, 0.0012±0.00005, and 0.0080±0.0050 for B1, B2, B3, and B4, respectively (p<0.01). Abrasion volume per chewing cycle (μm³/cycle) was 17650.8±9682.9, 27263.4±24746.8, 11836.5±4200.8, and 70436.8±73602.5 for B1, B2, B3, and B4, respectively (p=0.02).

Conclusion: The bonding strength and wear resistance of 3D printed denture materials vary by manufacturer. Formlabs Denture Base with Ivoclar Ivotion showed the highest fracture load, indicating superior bonding strength. In wear analysis, Ivoclar Ivotion PMMA Milled Teeth exhibited the least vertical wear and abrasion volume but had the highest failure rate under cyclic loading. While printed denture materials excel in bonding strength, their wear resistance may not be as good as milled denture teeth, highlighting the need to balance these properties in clinical applications.

## Introduction

Over the past decade, the integration of additive manufacturing, or 3D printing, into dentistry and dental laboratories has revolutionized removable prosthodontics. Initially, 3D printing was exclusively utilized in large dental laboratories and by dental manufacturers. However, the introduction of the cost-effective yet high-precision Form 2 printer by Formlabs [1] made in-office 3D printing commercially viable for dental offices and small dental laboratories. This advancement has empowered regular dentists, small dental laboratories, and dental schools to produce and fabricate dental casts, implant surgical guides, occlusal splints, and other intraoral devices and removable prostheses on-site [1]. This transformation has been driven by the rapid emergence of cost-effective yet highly accurate in-office 3D printers [1]. Initially, 3D printing applications in prosthodontics focused on static implant-guided surgery [1], but the technology quickly expanded to include the fabrication of occlusal splints [2], removable dentures [3], even guided appliances for soft tissue surgery [4], as well as orthodontic retainers and aligners [5]. The number of estimated edentulous patients in the United States increased from 31 million in 1991 to 38 million in 2020 [6]. This increase, along with the rising costs of conventional denture fabrication and a decrease in the number of removable laboratory technicians, has rapidly pushed the industry toward the digital fabrication of complete dentures [3]. 

In dentistry, resins used for 3D printing are hybrid materials that undergo UV light polymerization and consist of a combination of a base, photoinitiators, stabilizers, and pigments [7]. Typically, the base includes monomers or oligomers such as epoxy and acrylate monomers, although vinyl ether monomers and other types can also be used [8,9]. To create complete dentures via 3D printing, the denture base is printed first, followed by attaching either prefabricated denture teeth, usually a composite of polymethyl methacrylate (PMMA) and other filled resins, or 3D printed denture teeth using a bonding agent. Earlier studies have demonstrated that the bond strength and fracture toughness between 3D printed denture bases and artificial teeth are lower compared to traditional PMMA denture bases and those produced through subtractive digital manufacturing with prefabricated artificial teeth [10] However, other studies suggest that the bond strength between 3D printed denture tooth and base materials can be optimized with appropriate bonding protocols [11,12].

The rapid introduction of new 3D printed materials for denture bases and, more recently, denture teeth presents great opportunities for practitioners and laboratories. These innovations streamline denture manufacturing, reduce fabrication costs, address the shortage of conventional dental laboratory technicians, and promise clinical outcomes equal to or superior to conventional materials [13-15]. However, they also bring new challenges, particularly in developing expertise and conducting research to validate the clinical suitability and biological [16] and physical properties of these new materials compared to conventional PMMA denture base resin [12,17-19]. When a new denture tooth material is introduced, two critical questions for clinicians are whether the material bonds efficiently to current denture base materials and whether it has comparable wear resistance to conventional denture tooth materials.

Conventionally, the denture teeth are highly processed and often cross-linked PMMA and/or composite resin materials that are bonded to the conventional PMMA denture base materials. Most current light polymerization 3D printed teeth have to be bonded to the light polymerized denture bases. This study investigates a new 3D printed denture tooth material, Premium Teeth (Formlabs). The first hypothesis is that the Premium Teeth resin provides similar bond strength to 3D printed denture bases when bonded with different 3D printed denture base resins and adhesives. The second hypothesis is that the Premium Teeth resin has similar wear resistance, measured by vertical height and volumetric losses, compared to conventional denture teeth.

## Materials and methods

Denture tooth-denture base bonding analysis

For the denture tooth-denture base bonding analysis, Premium Teeth from Formlabs were used as the denture tooth material. Three different denture base-bonding protocols were employed (Table 1), each with six specimens (n=6 per group). Specimens were prepared, and the sample size was determined following the ISO-TS-19736-2027 standard. This involved positioning a 3D printed tooth, mimicking a central incisor that was then bonded to the 3D printed denture base. The 3D printers and post-processing methods were performed based on specific manufacturer's recommendations (Table 1). All tooth specimens were printed using the Premium Teeth (Formlabs). The tooth specimen was then bonded to the two types of base specimens, Lucitone Digital Print (Dentsply) or DENTCA (which is also the recommended denture base material for Formlabs), using three bonding systems, Lucitone Fuse (Dentsply), Formlabs Denture Base (Formlabs), and Ivoclar Ivotion Bonding. The group assignment and details are shown in Table 1. Group A1 utilized the Lucitone Digital Print-3D Denture Base bonded with the Lucitone Fuse System from Dentsply, Group A2 used the Formlabs Denture Base bonded with the Formlabs Denture Base Bonding System, and Group A3 used the Formlabs Denture Base bonded with the Ivoclar Ivotion Bonding System from Ivoclar.

**Table 1:** Specimen description and grouping for bonding analysis

	Group A1	Group A2	Group A3
Tooth material	Premium Teeth	Premium Teeth	Premium Teeth
Tooth printer	Formlabs Form 3B	Formlabs Form 3B	Formlabs Form 3B
Wash+cure	Formlabs Wash Formlabs Cure	Formlabs Wash Formlabs Cure	Formlabs Wash Formlabs Cure
Base material	Lucitone Digital Print-3D Denture Base	DENTCA Denture Base (Formlabs Denture Base)	DENTCA Denture Base (Formlabs Denture Base)
Base printer	ASIGA UV Max	Formlabs Form 3B	Formlabs Form 3B
Post-processing	Formlabs Wash Lucitone Digital Cure	Formlabs Wash Formlabs Cure	Formlabs Wash Formlabs Cure
Bonding system	Lucitone Fuse	Formlabs Denture Base	Ivoclar Ivotion Bonding
Number	6	6	6

After preparation, the bonded specimens were subjected to a palatal load at the incisal region at a 90° angle from the tooth's long axis until failure. Fracture surfaces were examined under 10× magnification to classify failure modes. Statistical significance in bond strength between groups was determined using ANOVA with α=0.05.

Wear analysis

For the wear analysis (Table 2), the same denture base material (Lucitone Digital Print-3D Denture Base) and bonding system (Lucitone Fuse System, Dentsply) were used. Four different denture tooth materials were tested, each with eight specimens (n=8 per group): Group B1 used Formlabs Premium Teeth, Group B2 used SprintRay High Impact Denture Teeth, Group B3 used Lucitone Digital IPN Premium Tooth, and Group B4 used Ivotion PMMA Milled Teeth (Ivoclar). Specimens were prepared similarly to the bonding analysis, with a denture tooth vertically positioned using modeling wax and fastened to a wax cylinder. A silicone mold replicated the wax assembly, and the denture tooth and the denture base were placed in the mold and bonded.

**Table 2:** Specimen description and grouping for wear analysis

	Group B1	Group B2	Group B3	Group B4
Teeth material	Premium Teeth	SprintRay High Impact Denture Teeth	Lucitone Digital IPN Premium Tooth	PMMA Milled Teeth (Ivoclar Ivotion Dent)
Teeth printer	Formlabs Form 3B	SprintRay Pro95	Formlabs Form 3B	N/A
Wash+cure	Formlabs Wash Formlabs Cure	Formlabs Wash SprintRay Cure	Formlabs Wash Lucitone Digital Cure	N/A
Base material	Lucitone Digital Print-3D Denture Base	Lucitone Digital Print-3D Denture Base	Lucitone Digital Print-3D Denture Base	Lucitone Digital Print-3D Denture Base
Base printer	ASIGA UV Max	ASIGA UV Max	ASIGA UV Max	ASIGA UV Max
Wash+cure	Formlabs Wash Lucitone Digital Cure	Formlabs Wash Lucitone Digital Cure	Formlabs Wash Lucitone Digital Cure	Formlabs Wash Lucitone Digital Cure
Bonding system+associated IFU	Lucitone Fuse System	Lucitone Fuse System	Lucitone Fuse System	Lucitone Fuse System
Number	8	8	8	8

These bonded specimens underwent a chewing simulation with cyclic loading of 1,200,000 cycles to mimic five years of masticatory function [20]. Sample failures were recorded, noting the number of cycles each specimen endured before failure. Vertical wear and abrasion volumes were measured to assess wear resistance. Vertical height loss was recorded in millimeters, and abrasion volume was documented in cubic millimeters. ANOVA with α=0.05 determined statistical differences in wear resistance among the groups.

In summary, this study analyzed both the bonding strength and wear resistance of 3D printed denture materials. The bond strength was assessed by measuring the fracture load to failure for Groups A1, A2, and A3, while wear resistance was evaluated by documenting the failure rates, vertical wear, and abrasion volumes for Groups B1, B2, B3, and B4. Statistical analyses using ANOVA with α=0.05 provided insights into the performance differences among the tested materials, offering valuable information on their clinical suitability.

## Results

Bonding strength analysis

The fracture load to failure (Table 3) for the different bonding protocols showed statistically significant differences among the groups (p=0.94). Group A1, which used the Lucitone Digital Print-3D Denture Base with the Lucitone Fuse System, had a fracture load to failure value of 175±106 N. Group A2, bonded with the Formlabs Denture Base using the Formlabs Denture Base Bonding System, had a fracture load to failure value of 167±46.3 N. Group A3, which utilized the Formlabs Denture Base with the Ivoclar Ivotion Bonding System, exhibited a fracture load to failure value of 183±48.9 N. 

**Table 3:** Description of fracture analysis* Group A1 utilized the Lucitone Digital Print-3D Denture Base bonded with the Lucitone Fuse System from Dentsply, Group A2 used the Formlabs Denture Base bonded with the Formlabs Denture Base Bonding System, and Group A3 used the Formlabs Denture Base bonded with the Ivoclar Ivotion Bonding System from Ivoclar * in newton (N) **Fracture mode presented as a number of samples with mixed (M) or cohesive (C) ANOVA p=0.94

Specimen number	Group A1	Group A2	Group A3
1	139	133	218
2	361	146	94.3
3	240	154	200
4	117	224	229
5	88.1	121	164
6	103	227	191
Mean	174.68	167.50	182.72
Standard deviation	106.03	46.32	48.83
Fracture mode**	5M	4M/1C	3M/3C

The failure characteristics of the bonded specimens varied among the groups. Most failures were mixed in nature, indicating a combination of adhesive and cohesive failure. Notably, in Group A2, one specimen showed cohesive failure. In Group A3, half of the specimens exhibited cohesive failure, suggesting a stronger bond within the material itself compared to the adhesive interface.

Absolute values of height loss and abrasion volume do not necessarily correlate, as height loss considers only the vertical component, whereas abrasion volume encompasses all three dimensions. Given that not all specimens underwent the same number of chewing cycles, an additional analysis was conducted to provide the average abrasion volume and height loss per specimen per chewing cycle. This approach allows for a more accurate comparison of the results.

Wear resistance analysis

**Figure 1:**
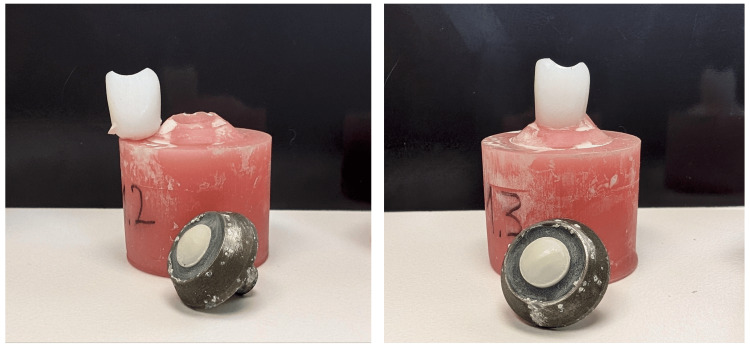
Samples from wear analysis Left: the failed sample after wearing analysis. Right: the intact sample after wearing analysis

In the cyclic loading test, only Group B4, which used Ivotion PMMA Milled Teeth, showed complete failure of all specimens within the 1,200,000 cycles. Groups B1 (Formlabs Premium Teeth), B2 (SprintRay High Impact Denture Teeth), and B3 (Lucitone Digital IPN Premium Tooth) had four, three, and five sample failures, respectively (Table 4). In terms of sample failure after cyclic loading, all 3D printed denture teeth were superior compared to the PMMA milled denture teeth. Figure 1 demonstrates two examples of wear resistance experiments with and without failure.

**Table 4:** Sample failures after chewing simulation* Group B1 used Formlabs Premium Teeth, Group B2 used SprintRay High Impact Denture Teeth, Group B3 used Lucitone Digital IPN Premium Tooth, and Group B4 used Ivotion Polymethyl Methacrylate Milled Teeth (Ivoclar) *The number of chewing simulation cycles that the specimen fails is shown. None means that the specimen did not fail after 1,200,000 cycles

Specimen	Group B1	Group B2	Group B3	Group B4
1	108,150	476,171	None	182,100
2	765,700	None	None	82,787
3	None	None	1,039,400	82,787
4	None	None	826,800	182,100
5	189,578	20,450	933,600	82,787
6	None	294,300	757,600	182,100
7	None	None	650,700	82,787
8	476,171	None	None	182,100

Vertical wear measurements revealed significant differences between the groups (p<0.01). Group B1 exhibited a vertical wear of 0.93±0.34 mm, Group B2 had 1.22±0.37 mm, Group B3 showed 1.05±0.27 mm, and Group B4 had the least wear with 0.37±0.02 mm (Table 5 and Table 6). Milled PMMA denture teeth demonstrated significantly less vertical wear compared to all 3D printed ones. There was no statistical difference in vertical wear among the 3D printed denture teeth.

**Table 5:** Description of vertical wear analysis Group B1 used Formlabs Premium Teeth, Group B2 used SprintRay High Impact Denture Teeth, Group B3 used Lucitone Digital IPN Premium Tooth, and Group B4 used Ivotion Polymethyl Methacrylate Milled Teeth (Ivoclar) ANOVA for all groups: p<0.01 ANOVA for Groups B1, B2, and B3: p=0.24

Specimen	Group B1 (mm)	Group B2 (mm)	Group B3 (mm)	Group B4 (mm)
1	0.61	1.36	1.04	0.35
2	1.27	0.30	1.27	0.20
3	0.75	1.37	1.07	0.27
4	0.47	1.26	1.34	0.59
5	0.73	1.33	1.07	0.34
6	1.33	1.37	0.97	0.69
7	1.30	1.46	1.16	0.06
8	1.00	1.31	0.45	0.41
Mean	0.93	1.22	1.05	0.36
Standard deviation	0.34	0.38	0.27	0.20

**Table 6:** Vertical height loss per cycle* Group B1 used Formlabs Premium Teeth, Group B2 used SprintRay High Impact Denture Teeth, Group B3 used Lucitone Digital IPN Premium Tooth, and Group B4 used Ivotion Polymethyl Methacrylate Milled Teeth (Ivoclar) *Wear per cycle ×10-4 µm per cycle ANOVA for all groups: p<0.01 ANOVA for Groups B1, B2, and B3: p=0.22

Specimen	Group B1 (×10^-4^ µm)	Group B2 (×10^-4^ µm)	Group B3 (×10^-4^ µm)	Group B4 (×10^-4^ µm)
1	58.00	12.00	9.00	34.00
2	19.00	33.00	11.00	84.00
3	6.00	12.00	11.00	131.00
4	4.00	11.00	16.00	43.00
5	42.00	67.00	13.00	76.00
6	11.00	83.00	14.00	47.00
7	11.00	13.00	18.00	177.00
8	25.00	11.00	4.00	48.00
Mean	22.00	30.25	12.00	80.00
Standard deviation	19.03	28.90	4.34	50.20

Absolute values of height loss and abrasion volume do not necessarily correlate, as height loss considers only the vertical component, whereas abrasion volume encompasses all three dimensions. Given that not all specimens underwent the same number of chewing cycles, an additional analysis was conducted to provide the average abrasion volume and height loss per specimen per chewing cycle. This approach allows for a more accurate comparison of the results.

Abrasion volume measurements also demonstrated variability among the groups (Table 7). Group B1 had an abrasion volume of 9.5±3.7 mm³, Group B2 had 12.2±4.7 mm³, Group B3 had 10.6±3.5 mm³, and Group B4 showed a significantly lower abrasion volume at 2.2±1.3 mm³ (p<0.01).

**Table 7:** Abrasive volume loss Group B1 used Formlabs Premium Teeth, Group B2 used SprintRay High Impact Denture Teeth, Group B3 used Lucitone Digital IPN Premium Tooth, and Group B4 used Ivotion Polymethyl Methacrylate Milled Teeth (Ivoclar) ANOVA for all groups: p<0.01 ANOVA for Groups B1, B2, and B3: p=0.24

Specimen	Group B1 (mm³)	Group B2 (mm³)	Group B3 (mm³)	Group B4 (mm³)
1	3.70	15.20	10.60	1.70
2	11.60	2.60	15.90	1.00
3	9.50	16.60	10.70	3.60
4	11.80	16.40	13.50	2.00
5	4.40	9.60	11.90	2.50
6	11.90	13.10	8.70	1.80
7	13.90	14.50	9.60	0.70
8	9.50	9.50	4.00	4.40
Mean	9.54	12.19	10.61	2.21
Standard deviation	3.67	4.76	3.51	1.26

The height loss measurement from the final measuring cycle of each specimen was used as a reference (Table 7). The recorded height losses and abrasion volumes were then divided by the number of the last measuring cycle (Table 8). It is important to note that the final measuring cycle is not necessarily the same as the fracture cycle, which may occur due to factors such as the loosening of the specimen before the actual fracture. 

**Table 8:** The last cycle used to measure volume loss* Group B1 used Formlabs Premium Teeth, Group B2 used SprintRay High Impact Denture Teeth, Group B3 used Lucitone Digital IPN Premium Tooth, and Group B4 used Ivotion Polymethyl Methacrylate Milled Teeth (Ivoclar) *The fracture cycle refers to the chewing cycle during which the fracture was visually confirmed by the examiner. The specimens not fractured were at 1,198,800 cycles

Specimen	Last measuring cycle number of each specimen			
	Group B1	Group B2	Group B3	Group B4
1	105,600	1,159,200	1,198,800	105,600
2	672,000	93,600	1,198,800	24,000
3	1,198,800	1,159,200	969,600	20,400
4	1,198,800	1,159,200	817,200	138,000
5	172,800	199,200	832,800	44,400
6	1,198,800	164,400	704,400	146,400
7	1,198,800	1,159,200	639,600	3,600
8	393,600	1,159,200	1,198,800	85,200

The abrasion volume per chewing cycle further highlighted these differences (Table 9). Group B1 had an abrasion volume of 17650.8±9682.9 μm³/cycle, Group B2 had 27263.4±24746.8 μm³/cycle, Group B3 had 11836.5±4200.8 μm³/cycle, and Group B4 showed a significantly higher volume at 70436.8±73602.5 μm³/cycle (p=0.02).

**Table 9:** Volume loss per chewing cycle ANOVA for all groups: p=0.02 ANOVA for Groups B1, B2, and B3: p=0.16

Specimen	Abrasion volume per chewing cycle (µm³/cycle)			
	Group B1	Group B2	Group B3	Group B4
1	34848	13138	8825	16288
2	17262	28098	13272	39583
3	7925	14329	11025	177941
4	9835	14130	16532	14130
5	25694	48293	14277	57207
6	9885	79440	12394	12022
7	11570	12491	15072	194444
8	24187	8187	3295	51878
Mean	17,650.75	27,263.25	11,836.50	70,436.63
Standard deviation	9,682.62	24,746.67	4,200.83	73,602.40

In summary, the bonding strength analysis revealed that the bonding protocols influenced the fracture load to failure, with Group A3, Formlabs Premium Teeth with Ivoclar Ivotion Bonding, exhibiting the highest bond strength. The wear resistance analysis indicated that while the Ivotion PMMA Milled Teeth (Group B4) had the least vertical wear and abrasion volume compared to 3D printed systems, the PMMA milled teeth were more prone to failure under cyclic loading.

## Discussion

Since the introduction of additive manufacturing to dentistry, in particular the fabrication of removable prostheses, there has been an exponential development of 3D printed materials for denture bases and denture teeth. These new materials were advocated for clinical applications; however, there has been a clear lack of preliminary research prior to the materials being introduced to the market. Thus, clinicians and laboratory technicians may recommend or utilize the material with any scientific support. Specific to new materials for denture teeth, two important research questions are (1) if the new denture tooth material bonds as well as the existing denture base material and (2) if the new denture tooth material has sufficient wear resistance. Thus, this study aimed to elucidate these two research questions with regard to the new 3D printed denture material, Premium Teeth (Formlabs), by examining the bonding strength to an existing 3D printed denture base materials and their wear resistance subjected to chewing simulation. The results demonstrated that the choice of bonding protocol and material significantly affects the performance of the denture assemblies.

For the bonding strength analysis, three group assignments with the same Premium Teeth (Formlabs) were done to account for the bonding of the new denture tooth material for the existing 3D printed base and bonding system (Group A1), the bonding of the new denture tooth material with the recommended base material and recommended bonding system (Group A2), and the bonding of the recommended base material and a generic bonding system (Group A3). The fracture load to failure values for the groups bonded using different protocols (A1, A2, and A3) do not have statistically significant differences; thus, the null hypothesis was quantitatively accepted. However, the qualitative analysis showed some differences in the mode of failure. Group A3, bonded with the Formlabs Denture Base using the Ivoclar Ivotion Bonding System, exhibited the highest fracture load (183±48.9 N), suggesting superior bonding strength. Also, the fact that about half of the failures were cohesive and the other half were mixed indicates that the Ivoclar Ivotion Bonding System may provide a better bonding between 3D printed denture teeth and bases compared to the other tested systems including the recommended Formlabs bonding or the one recommended with Dentsply base. These shear bond strengths are similar to the bonding of autopolymerized PMMA teeth to other materials. A recent study demonstrated a shear bond strength of 162.5±4.4 N when bonding an autopolymerized PMMA denture tooth to a polycarbonate denture base [21]. The shear bond strengths of 3D printed denture tooth to conventional denture tooth with 3D printed denture base in this study were similar to another previous study comparing the bond strength of a heat-process denture base, milled denture base, and 3D printed denture base with conventional denture tooth. The highest shear bond strengths of 180±26.76 N between the conventional denture teeth and heat-polymerized denture base and 108±12.98 N between the 3D printed base (Carbon Lucitone Digital Print, Dentsply) and conventional denture tooth were reported [22]. The average range of ~167-182 N shear bond strength of the Premium Teeth (Formlabs) to 3D printed denture base suggests that the new material's performance in terms of bonding to the denture base is comparable to other currently used systems. The mixed/cohesive failure examination, however, suggests that the bonding of the Premium Teeth (Formlabs) material is better with the recommended base but with the Ivoclar Ivotion Bonding System.

The wear resistance of the denture teeth materials was evaluated through cyclic loading. PMMA milled teeth (Group B4) was the only group where all specimens failed within the 1,200,000 cycles, indicating a lower durability under prolonged cyclic loading compared to 3D printed groups (B1, B2, and B3). However, PMMA milled teeth exhibited the least vertical wear (0.37±0.02 mm) and abrasion volume (2.2±1.3 mm³) compared to the 3D printed teeth. 3D printed teeth showed higher vertical wear and abrasion volumes but better durability under cyclic loading. While this study employed very high values of a simulated chewing cycle of 1,200,000 comparable to five years of functional mastication, other studies examined the lower cycle. A recent study that examined the wear of 3D printed teeth (NextDent C&B MFH Micro Filled Hybrid, 3D Systems Corporation) compared to prefabricated denture teeth with 60,000 chewing simulation cycles and 10,000 thermocycles suggested that the 3D printed teeth had lower wear resistance compared to the prefabricated one [23]. While our results demonstrated that the 3D printed denture teeth had lower wear resistance than the prefabricated denture teeth made of high cross-linked PMMA/composite resin, another study showed that 3D printed teeth (DENTCA denture tooth resin; DENTCA, Inc.) subjected to 49 N for 30,000 cycles under thermocycling conditions in a mastication simulator exhibited a wear pattern similar to prefabricated PMMA denture teeth when opposed by metal and zirconia materials [24]. These differences may be attributed to the type of prefabricated denture teeth, the percentages of cross-links, and the ratio of composite resin to PMMA. When comparing 3D printed denture teeth with prefabricated low- and high-cross-linked PMMA denture teeth, the 3D printed one (Denture Teeth, Formlabs) had better wear resistance compared to PMMA denture teeth [25]. Opposing dentition or restorative materials may play an important role in the wear of denture tooth materials. While modern 3D printed tooth materials have the advantage of fracture durability, there is room for improvement in terms of long-term wear resistance. Clinicians should carefully evaluate the risks and benefits of using 3D printed denture teeth, milled denture teeth, or conventional denture teeth, considering each patient's unique oral function and needs. Factors such as potential wear or tooth debonding from opposing dentition, type of prosthesis, parafunctional habits, and other individual characteristics should be taken into account.

The detailed analysis of vertical height loss and abrasion volume per chewing cycle provided additional insights. The differences in height loss and abrasion volume among the groups highlight the varying wear resistance properties of the materials. Group B4's higher abrasion volume per chewing cycle (70436.8±73602.5 μm³/cycle) indicates that although its initial wear resistance is high, it might degrade more rapidly once wear begins. This finding underscores the importance of balancing initial wear resistance with long-term durability. In addition to this, it is possible that the intraoral environment may be even more hasher. Thermocycling, wet-dry cycling, as well as exposure to various pH in future studies may be a better measure for the oral environment and may be needed [7,22,24].

This study has several limitations. The sample size for each group was relatively small, which may affect the generalizability of the results. Additionally, the in vitro nature of the study may not fully replicate the complex conditions within the oral environment. Future research should include larger sample sizes and in vivo studies to validate these findings. Further research is also needed to explore the long-term clinical performance of these materials in various patient populations and to develop new formulations that combine strong bonding properties with enhanced wear resistance. Additionally, other important properties of these new 3D printed materials, such as long-term clinical color stability and resistance to material degradation, have not been extensively tested. The long-term color stability of dental materials is crucial for maintaining aesthetic outcomes over time, as discoloration can lead to patient dissatisfaction and the need for premature replacement. Similarly, resistance to material degradation is vital for ensuring the durability and functional longevity of dental prostheses. Factors such as exposure to oral fluids, varying pH levels, and mechanical stress can contribute to the degradation of dental materials. Future studies should prioritize evaluating these properties under simulated oral conditions to provide a comprehensive understanding of the clinical performance and longevity of 3D printed dental materials. This will help practitioners make informed decisions and improve the quality of care provided to patients. Understanding the interplay between different material properties will be crucial for advancing the field of 3D printed prosthodontics.

## Conclusions

The study demonstrated that the new 3D printed denture tooth material, Premium Teeth (Formlabs), has similar bonding strength and wear resistance as contemporary 3D printed denture tooth materials. However, the Ivoclar Ivotion Bonding System provides the best 3D printed denture tooth to 3D printed denture base. When compared to a milled PMMA material, the 3D printed denture teeth have lower wear resistance under cyclic loading; however, the 3D printed ones have better fracture resistance. Balancing these properties is essential for optimizing the clinical performance of 3D printed dentures. Clinicians should also be mindful of other disadvantages of 3D printing denture teeth such as color stability, bonding capacity to conventional PMMA material, and possibly biological effects from remaining unpolymerized resins. Ongoing research and innovation are necessary to enhance the durability and functionality of these new 3D printed materials in clinical applications.
